# Investigating the Relationship between Cerebrospinal Fluid and Magnetic Induction Phase Shift in Rabbit Intracerebral hematoma expansion Monitoring by MRI

**DOI:** 10.1038/s41598-017-11107-1

**Published:** 2017-09-11

**Authors:** Mingsheng Chen, Qingguang Yan, Jian Sun, Gui Jin, Mingxin Qin

**Affiliations:** 1 College of Biomedical Engineering, Third Military Medical University, Chongqing, 400038 China; 2 State Key Laboratory of Trauma, Burns and Combined Injury, Institute of Surgery Research, Daping Hospital, Third Military Medical University, Chongqing, 400042 China

## Abstract

In a prior study of intracerebral hemorrhage monitoring using magnetic induction phase shift (MIPS), we found that MIPS signal changes occurred prior to those seen with intracranial pressure. However, the characteristic MIPS alert is not yet fully explained. Combining the brain physiology and MIPS theory, we propose that cerebrospinal fluid (CSF) may be the primary factor that leads to hematoma expansion being alerted by MIPS earlier than with intracranial pressure monitoring. This paper investigates the relationship between CSF and MIPS in monitoring of rabbit intracerebral hemorrhage models, which is based on the MIPS measurements data, the quantified data on CSF from medical images and the amount of injected blood in the rabbit intracerebral hemorrhage model. In the investigated results, a *R* value of 0.792 with a significance of 0.019 is observed between the MIPS and CSF, which is closer than MIPS and injected blood. Before the reversal point of MIPS, CSF is the leading factor in MIPS signal changing in an early hematoma expansion stage. Under CSF compensation, CSF reduction compensates for hematoma expansion in the brain to keep intracranial pressure stable. MIPS decrease results from the reducing CSF volume. This enables MIPS to detect hematoma expansion earlier than intracranial pressure.

## Introduction

Intracerebral hemorrhage (ICH) is highly prevalent all over the world and carries high mortality rates. Continuous monitoring of ICH is very important for clinical diagnosis and treatment^1^. The intracranial pressure (ICP) measurement is currently the most popular ICH monitoring method. However, the ICP measurement is an invasive method, which requires the introduction of a catheter into the brain. This invasive method has the disadvantage of possibly causing infection or secondary hemorrhage, discomfort and pain. To avoid the disadvantages of this invasive method, a non-invasive method is desired for clinical use.

**Figure 1:**
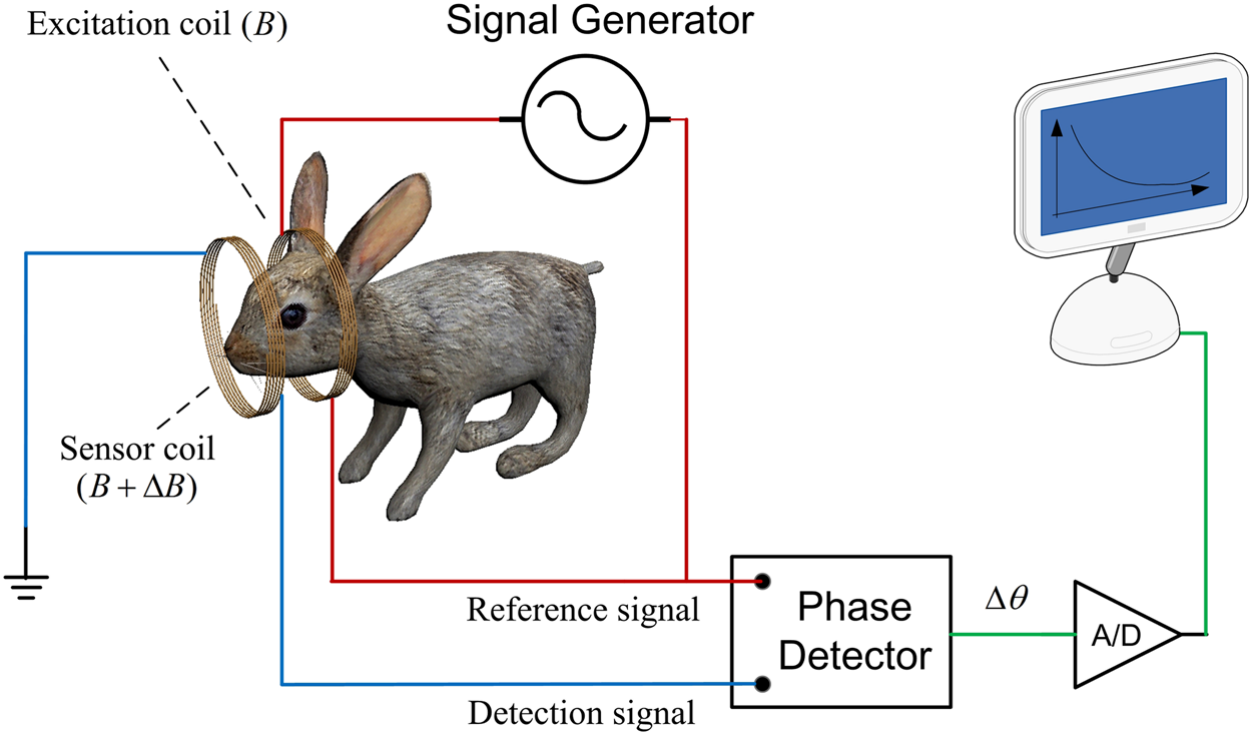
Block diagram of the MIPS experimental prototype.

Magnetic induction based monitoring is a new and emerging non-invasive method, which contains magnetic induction tomography (MIT) and magnetic induction phase shift (MIPS) technology^2^. MIT exhibits prospects the detection of cerebral hemorrhage and edema, but it also shows technical difficulties in the detection of biological tissues. Different from MIT, MIPS technology is consists of single excitation coil and single sensor coil and doesn’t need the heavy computational imaging algorithm. The MIPS experimental prototype of this paper is shown in Fig. 1. MIPS can be used non-invasively, is low-cost, fast, and can be a continuously bedside monitoring clinical method. ICH monitoring using MIPS is a very promising method, especially in the poor and rural district. Studies have been conducted to develop the MIPS techniques to apply in medical detection, such as reflecting the conductivity changes in the brain^3^, detecting hydrocephalus^4^, detecting and discriminating brain edema and hematoma^5–7^, detecting brain ischemia^8^, detecting cerebral hemorrhage in rabbits^9, 10^, and improving the performance of MIPS^11^. But most of the researches take the brain as a entirety in MIPS detection, investigation of impact of different brain tissues on MIPS signal is still not reported in current works.

When we investigated MIPS detection for intracerebral hematoma expansion in rabbits, we found that the MIPS signal is more sensitive than ICP during early hematoma expansion and can offer timely alerts to monitor ICH.

**Figure 2:**
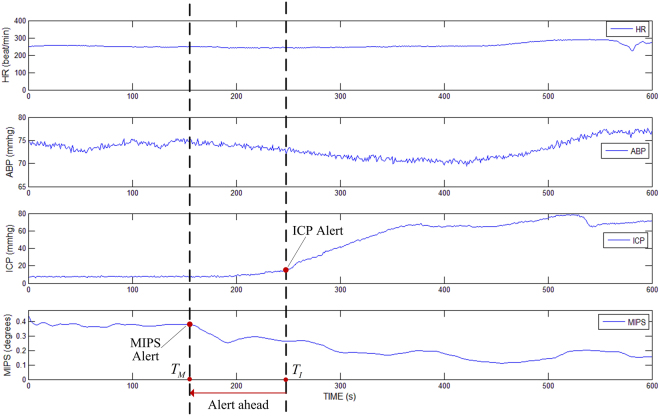
Experimental result of MIPS alert ahead of ICP in early hematoma expansion monitoring.

In experiments of the previous work, ten rabbits were detected and recorded synchronously by MIPS and ICP. In results of the ten rabbits, absolute values of first derivative of MIPS were greater than the absolute value of first derivative of ICP in early hematoma expansion, which meant MIPS changed more intensely than ICP in early stage of hematoma expansion. Figure 2 shows the experimental results of one of the rabbits monitored with heart rate, arterial blood pressure, ICP and MIPS. As the hemorrhage develops, the ICP start increasing from the critical point at *T*_*I*_, and the MIPS start decreasing at a critical point *T*_*M*_; however, the *T*_*M*_ characterized in MIPS detection occurs earlier in the process than the *T*_*I*_ characterized in ICP monitoring. Findings from the experiments are important in clinic. Compared with traditional ICP monitoring, it now seems possible that we can detect hematoma expansion in a timelier manner using MIPS and provide an earlier alert to doctors. The above experimental results raise the following questions: why do the MIPS indicate significant changes earlier than ICP during hematoma expansion and which tissue is involved as the primary factor in the brain that results in MIPS signal changing in hematoma expansion monitoring.

Combining brain physiology and MIPS detection theory, we propose that changes in the cerebrospinal fluid (CSF) may be the primary factor that hematoma expansion is detected earlier by the MIPS during ICH progression. Firstly, in the brain, CSF has a significantly higher conductivity than gray matter (GM), white matter (WM) and blood (at a frequency of 10.7 MHz, the conductivity of CSF is 6.69 times, 12.36 times, and 1.82 times higher than the conductivity of GM, WM or blood, respectively^12^). According to the magnetic induction theory, the MIPS readings are based on the conductivity of tissues between the excitation coil and the sensor coil. Secondly, during early hematoma expansion, the CSF compensation mechanism decreases CSF total volume in an attempt to maintain normal intracranial pressure. When this occurs, the ICP is maintained at a stable state, but the conductivity of the brain is changed, which could explain why MIPS is able to detect hematoma expansion earlier in the process than ICP can. The two facts support our hypothesis that CSF is the primary factor resulting in MIPS signal changing. However, these hypotheses need to be confirmed with experiments.

This paper investigates the relationship between the CSF and MIPS in hematoma expansion monitoring through experiments designed and performed on a rabbit ICH model. The study utilizes the medical imaging and available image processing techniques to observe and quantify changes in the quantity of CSF during hematoma expansion. Based on the quantified data on CSF from medical images, the relationship between the CSF and MIPS was investigated by analyzing the impact of the changed volumetric CSF on the MIPS signal.

## Results

The rabbits used in the MIPS measurements and MRI observation experiments were fed with uniform conditions by experimental animal center of the third military medical university. The selected rabbits weighed 2.2 ± 0.2 kg, and they had a mean head diameter measurement within a range of ±5%. Environmental conditions (temperature, humidity etc.) around the area where the experiments were conducted was tightly controlled. Finally, the MIPS measurement results for eight rabbits, observations on CSF and quantification results for all eight rabbits are included in the results presented here.

### MIPS measurement results

Table 1 shows the results of MIPS measurements on the eight ICH rabbits. Each measurement collected 2700 MIPS data points in 54 minutes. For comparison with the CSF observation, the data were smoothed on each 300 consecutive points and normalized.

**Table 1:** MIPS measurement results of the eight ICH rabbits.

Time (min)	Blood injection (ml)	rabbit 1	rabbit 2	rabbit 3	rabbit 4	rabbit 5	rabbit 6	rabbit 7	rabbit 8
6	0.33	100.2652	99.3756	99.7284	99.873	99.925	100.183	100.4718	100.4838
12	0.66	100.0558	98.1912	99.2334	99.171	99.171	99.7712	100.2348	100.233
18	1.00	99.48	97.2972	98.7932	98.778	98.778	99.3532	99.5864	99.7042
24	1.33	99.3584	96.927	98.6694	97.918	97.518	98.9064	99.4714	99.9792
30	1.66	99.2494	96.7842	98.4012	97.198	96.732	98.9564	99.547	100.417
36	2.00	99.507	97.0456	98.5262	97.772	96.832	98.9428	99.7418	100.6024
42	2.33	99.6194	97.2434	98.6388	97.405	97.333	98.685	100.315	101.0506
48	2.66	99.736	97.442	98.7586	98.817	97.986	99.218	100.016	101.0776
54	3.00	99.7768	97.83	98.7386	99.148	98.598	99.2944	100.3544	100.9496

### CSF observation and quantification results

**Figure 3:**
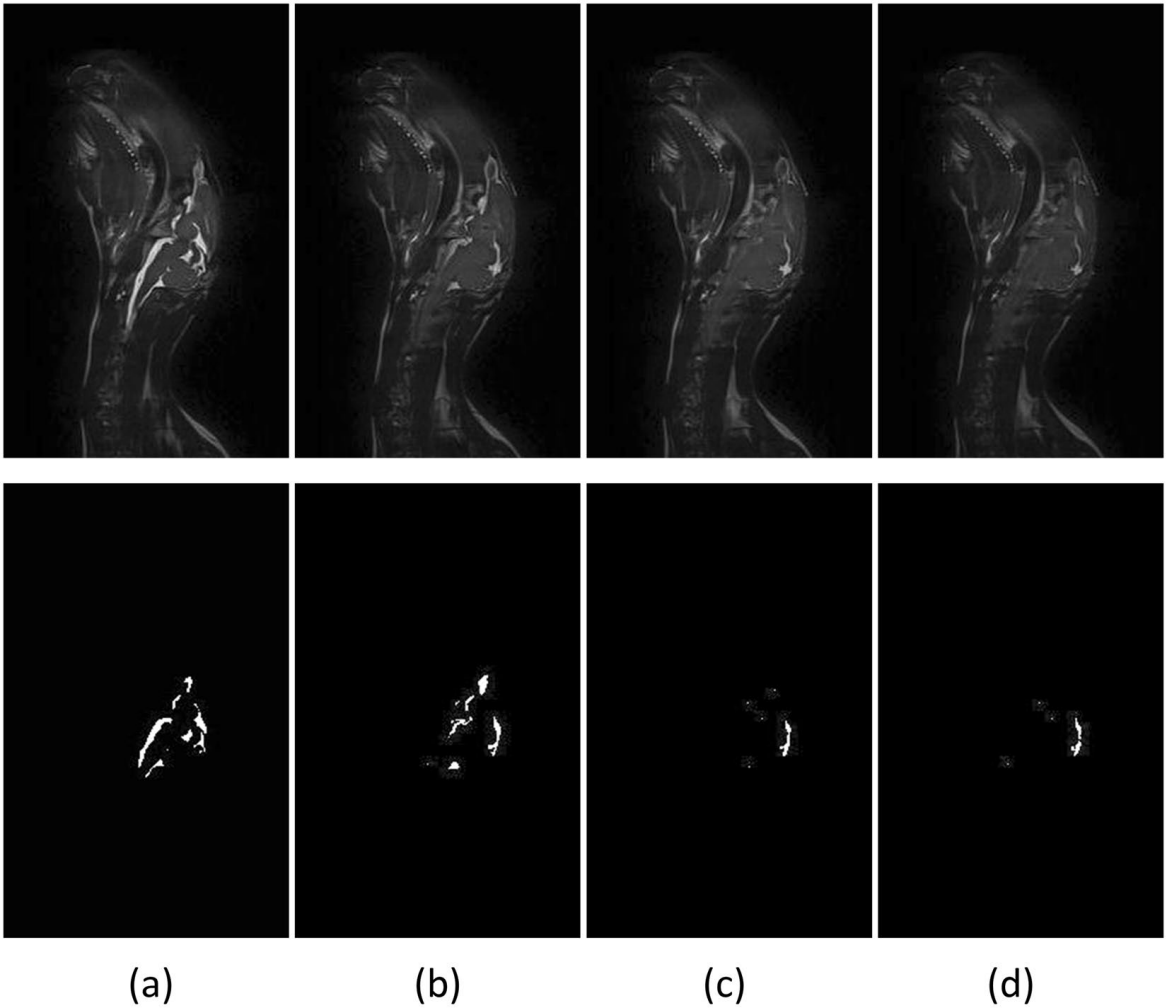
CSF observation experiments. Time points: (**a**) No blood injection, (**b**) 1 ml of blood injected, (**c**) 2 ml of blood injected and (**d**) 3 ml of blood injected.

**Figure 4:**
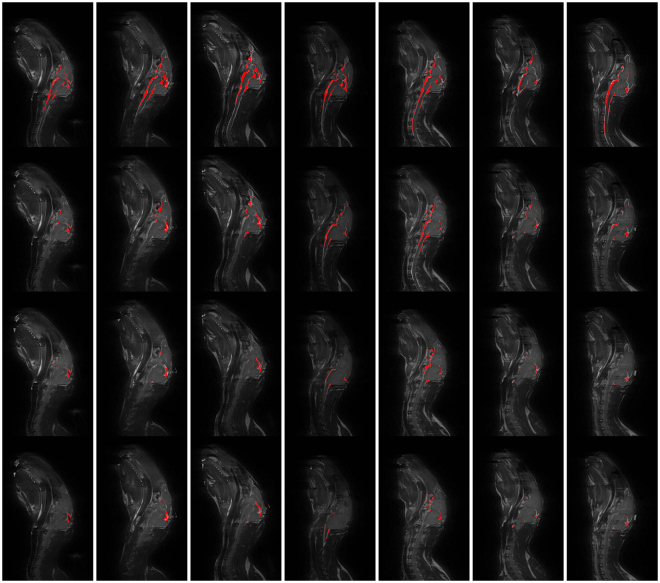
CSF observation experiment results from the seven rabbits.

Figure 3 shows the CSF imaging results for the first ICH rabbit on the 95th slice. The columns from one to four in the images correspond to rabbits treated as follows: no blood injection, 1 ml of blood injection, 2 ml of blood injection and 3 ml of blood injection. The first row shows the original images, and the second row shows the corresponding images after CSF extraction. Figure 3 shows that CSF decreases as the amount of injected blood increases. Figure 4 shows the CSF in the experimental results on the 95th slice in the other seven rabbits. The CSF extracted by our image processing algorithm is highlighted in red color. In the figure, each column corresponds to one rabbit. Rows one to four correspond to the results from rabbits treated as follows: no blood injection, 1 ml of blood injection, 2 ml of blood injection and 3 ml of blood injection.

**Figure 5:**
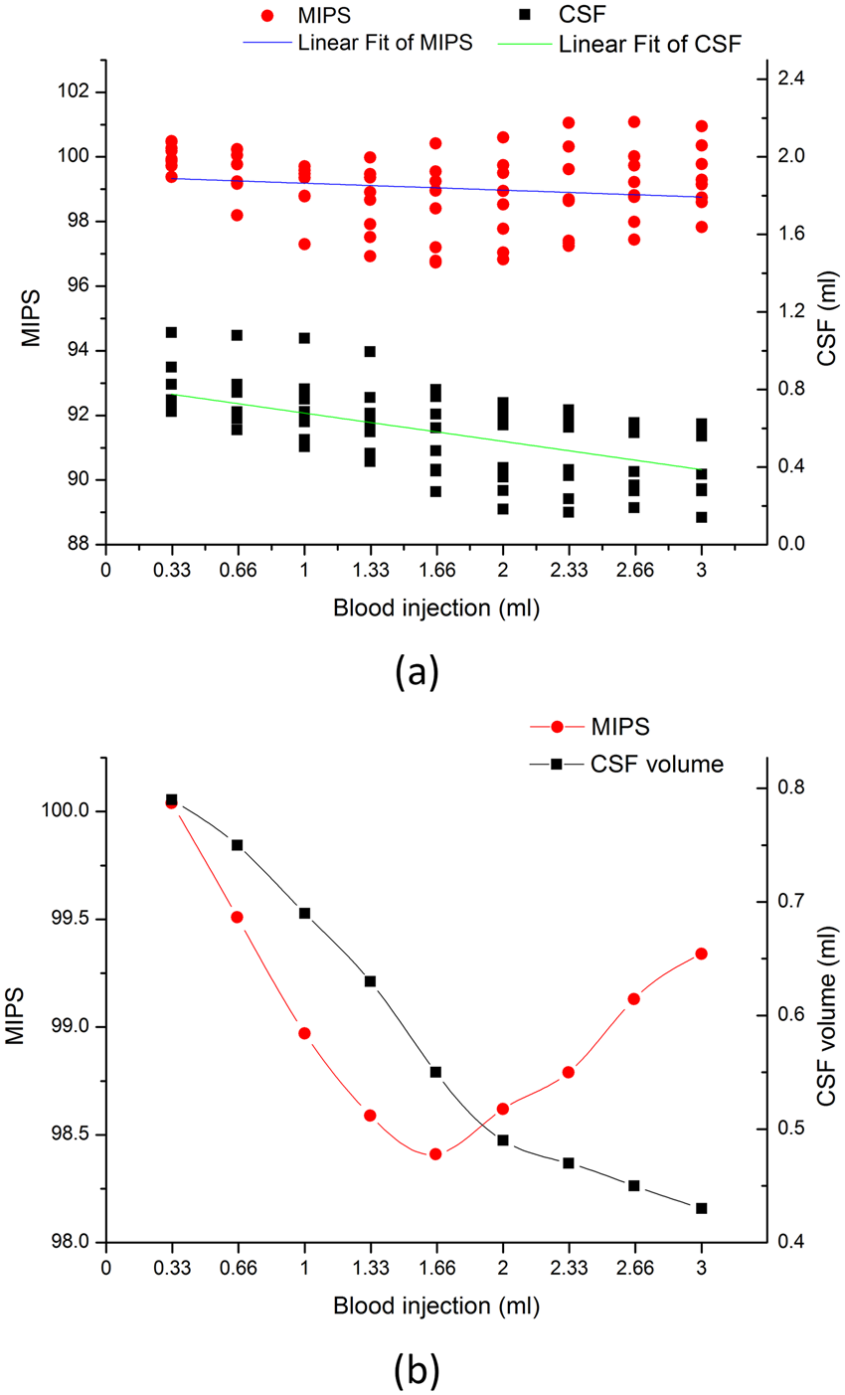
Experimental data summary. (**a**) Scatter plot of all collected MIPS and CSF volume data. (**b**) Curve plot of the mean MIPS and CSF volume for all rabbits.

Table 2 shows the results of CSF quantification of data from eight ICH rabbits. Figure 5 provides a visual summary of the experimental data. Figure 5(a) is a scatter plot of all collected MIPS and CSF volume data. Figure 5(a) shows the line fitting curves for the MIPS measurements and CSF quantification. A decreasing trend is seen in the two fitted lines indicating that the MIPS decrease is simultaneous with the decrease in the CSF volume. Figure 5(b) shows a curve plot of mean MIPS measurements and CSF volume quantification for all rabbits. As seen in the plot, the MIPS first decreases then increases at the point where approximately 1 ml~1.33 ml of blood were injected into the rabbit’s head.

**Table 2:** CSF quantification results for all eight ICH rabbits.

Time (min)	Blood injection (ml)	rabbit 1	rabbit 2	rabbit 3	rabbit 4	rabbit 5	rabbit 6	rabbit 7	rabbit 8
6	0.33	1.0949	0.9156	0.6936	0.6872	0.687	0.6962	0.7474	0.8276
12	0.66	1.0796	0.8273	0.5925	0.6856	0.6805	0.6604	0.6506	0.7862
18	1.00	1.0653	0.8041	0.5285	0.6861	0.6344	0.5058	0.5418	0.7504
24	1.33	0.9959	0.7591	0.4584	0.6781	0.638	0.4289	0.4715	0.5819
30	1.66	0.8001	0.7636	0.3889	0.6735	0.6028	0.2732	0.3792	0.4842
36	2.00	0.6769	0.7321	0.3485	0.6555	0.6171	0.1839	0.2794	0.3974
42	2.33	0.6751	0.6962	0.3565	0.636	0.6054	0.1674	0.2363	0.3875
48	2.66	0.6225	0.6296	0.3078	0.622	0.5782	0.191	0.2776	0.3766
54	3.00	0.5702	0.6239	0.2885	0.6219	0.5596	0.1408	0.278	0.3629

### Experimental data analysis

The CSF and injected blood are the primary variables in the brain of the ICH rabbit. The CSF and injected blood were investigated to determine the correlation with the MIPS. Tables 3 and 4 show the multiple correlations between MIPS, CSF and injected blood. The multiple correlation coefficient *R* is from 0 to 1. A value of *R* that is closer to 1 indicates that the CSF and injected blood are strongly correlated with the MIPS readings. In the statistical results shown in Tables 3 and 4, the *R* value is 0.824, and the regression significance is less than 0.05. These results indicate that the CSF and injected blood correlates significantly with the MIPS.

**Table 3:** Model summary of multiple correlation among MIPS, CSF and injected blood.

Model	R	R Square	Adjusted R Square	Std. Error of the Estimate
1	0.824^a^	0.680	0.573	0.33882

^a^Predictor variables: Injected Blood, CSF.

**Table 4:** Anova^a^ to determine multiple correlations between MIPS, CSF and injected blood.

model		Sum of Squares	df	Mean Square	F	**Sig**.
1	Regression	1.462	2	0.731	6.369	0.033^b^
	Residual	0.689	6	0.115		
	Total	2.151	8			

^a^Dependent variable: MIPS. ^b^Predictors: (constant), Injected Blood, CSF.

The correlation between the MIPS with CSF or injected blood was investigated. Table 5 shows the results of the partial correlation analysis of the relationship between the MIPS, CSF and injected blood. Firstly, the injected blood was set as the controlled variable, and the correlation between MIPS and CSF is analyzed. The partial correlation coefficient between MIPS and CSF showed a value of 0.792 with a significance level of 0.019, indicating that the MIPS is significantly correlated with CSF. Next, the CSF was set as the controlled variable, and the analysis results showed that the partial correlation coefficient between MIPS and injected blood was 0.748 with a significance level 0.033. The partial correlation coefficient between MIPS and CSF is stronger than the coefficient between MIPS and injected blood. This indicates that the MIPS is more relevant with the CSF than injected blood.

**Table 5:** Partial correlation analysis to determine the relationship between MIPS, CSF and injected blood.

Control Variable		CSF		Injected blood	
		R	Sig	R	sig
Injected Blood	**MIPS**	0.792	0.019		
CSF				0.748	0.033

**Figure 6:**
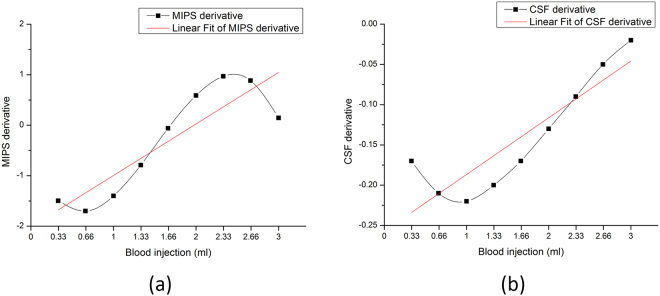
Derivative curve of MIPS measurements and CSF change. (**a**) Derivative curve of MIPS, (**b**) Derivative curve of CSF change.

Figure 6 shows that the derivative curve fits for changes in the MIPS and CSF. Figure 6(a) shows the derivative curve for the high class fitted MIPS measurements. Figure 6(b) shows the derivative curve for the high class fitted CSF change. The derivative curves reveal that the rate of change of the MIPS and CSF relative to the injected blood volumes. The red lines in (a) and (b) are the line fittings for the derivative curves. The two similar red lines reveal the consistent trend in the change rate for MIPS and CSF.

The correlation between the derivative curve of MIPS and CSF was also analyzed. A correlation coefficient *R* value of 0.751 was obtained with a significance level of 0.02. This finding indicates that the MIPS measurement is strongly correlated with CSF change, but also the MIPS change rate is strongly correlated with of CSF change rate. Furthermore, it illustrates the close correlation between MIPS measurement and CSF change.

## Discussion

MIPS is a potential brain monitoring technique, and it is low cost, accessible, and non-invasive. The people especially in the poor and rural district will be benefit from the using of MIPS measurements. MIPS can be utilized to distinguish edema and hematoma. However, the impact of brain tissues on MIPS signal is still unknown. In this paper, we report study findings on the relationship between CSF and MIPS in hematoma expansion monitoring. We posit that the CSF is the most contributory factor on MIPS but not bleeding in hematoma expansion monitoring. This hypothesis was based on our previous work and the fact that CSF has the highest conductivity among main brain tissues. Medical imaging and available image processing techniques were used to observe and quantify changes in the CSF in ICH rabbits. Based on the amount of injected blood and quantified cerebral data on CSF changes in medical images, the study investigated the relationship between MIPS and CSF during hematoma expansion monitoring by analyzing the impact of the CSF and blood injected in the brain on MIPS signals.

From Figs 3 and 5, we verified that the MIPS signals decrease concurrently with a decrease in the CSF volume at an early stage during hematoma expansion. The early stage CSF decrease is a result of the CSF compensation mechanism and demonstrates the possibility of an alert characteristic of the MIPS compared with ICP monitoring, which occurs later. It can be explained that the MIPS decreases along with CSF reducing while CSF compensation mechanism reduces CSF to keep ICP stable. Furthermore, changing the constituent ratio of tissues with different conductivity in the brain can result in changes in the MIPS signals. This finding has been validated using animal ICH models by comparing with previous validation from physical models^13, 14^.

The impact of the changing volumetric CSF and injected blood on the MIPS signal was investigated. Firstly, Tables 3 and 4 show the significant multiple correlation between MIPS, CSF and injected blood. The more interesting correlation of the MIPS with the CSF or injected blood was investigated by partial correlation analysis. The statistical analysis results in Table 5 shows that the MIPS is significantly correlated with the CSF and is more correlated with the CSF than the injected blood. The result is consistent with previously reported data in which the CSF shows a greater impact on the MIPS because of its significantly higher conductivity. The rates of change for the MIPS and CSF following blood injections in the rabbit brain show a similar trend, which is seen in fitted lines in Fig. 6. The change rates are also significantly correlated as shown through statistical analysis. Referring to Figs 5 and 6, the MIPS decreases as fast as the CSF decreases. When the CSF compensation mechanism fails, the CSF decreases slowly, and the MIPS also changes slowly at a uniform blood injection velocity during the early hematoma expansion stage. This outcome indicates the close relationship between the MIPS and CSF and also means that the MIPS signal reflects substantial information related to changes in CSF.

Another result in Fig. 5 indicates that there is a reversal point in the MIPS and an inflection point for the CSF volume. The reversal point is slightly ahead of inflection point. These observations are relevant to the CSF compensation mechanism. Firstly, the inflection point of the CSF volume corresponds to the time at which the CSF compensation begins losing efficacy. In the period under CSF compensation in hematoma expansion, the CSF is the leading impact factor on the MIPS signal because of its significantly higher conductivity, which was also verified in the experimental results in Table 5 and Fig. 6. When the proportion of the CSF in the brain decreases along with CSF compensation, the impact of the CSF on the MIPS also decreases, and it continues until the MIPS reverses. When CSF reducing is close to lose CSF compensation efficacy, it is hypothesized that the blood becomes leading impact factor on MIPS signal, which makes the MIPS elevation after the reversal point. The reversal point for the MIPS is important in signal processing. It means that the CSF is losing compensation efficacy and enters into a period of rapid growth of ICP. The zero-crossing point in the derivative curve for the MIPS in Fig. 6(a) can also be set as the feature point corresponding to the reversal of the MIPS.

The impact of the CSF on MIPS monitoring in hematoma expansion is important for MIPS research and in clinical practice. Based on the results in this paper, we propose that bleeding in the brain should not be regarded as the leading factor on the MIPS signal in hematoma expansion early stage monitoring. More attention should be paid to CSF in the brain detection methods based on brain tissue electrical property (EIT, MIT, etc.). The information about CSF can be used by doctors to monitor the physiological changes in the brain and hematoma expansion. In monitoring hematoma expansion using the MIPS, the reversal point of the MIPS is very important in that it indicates that the CSF compensation is losing efficacy and that the brain is losing protection and entering a period of rapid growth of ICP. Based on the investigation results, the MIPS also can be combined with ICP monitoring in clinical settings. If the ICP reading is close to normal and the MIPS decreases fast, it may indicate that the patient is still bleeding and experiencing CSF compensation. However, if the ICP is close to normal and the MIPS change at a slower rate, it may indicate that the bleeding is not having significant expansion. If the ICP is at a high level, a stable MIPS signal indicates that serious hematoma expansion has stopped while significant changes in the MIPS signal indicates that serious hematoma expansion is ongoing. And we can discern the part of the reversal curve of MIPS that the MIPS signal is on outside of overall trend to indicate the development of hematoma expansion. After smoothing and denoising, firstly computes the first derivative of MIPS and computes maximum of the first derivative of MIPS. Then the section after the maximum of one derivative is the section of the reversal curve that the MIPS signal is on outside of overall trend.

Except the two most contributory factors (CSF and blood), edema can also change the electric property of tissues. But on ICH model in rats, edema surrounding the hemorrhage appeared after 4 hours from hematoma expansion beginning, which peaks at 24 hours and fades away from 5 days^15^. In our work, the ICH model in rabbits were injected 3 ml autologous blood within 54 minutes. The time is not enough for the formation of marked brain edema. Meanwhile, as the MRI scanning result on rabbit head with 3 ml blood injected by SPACE sequence is shown in Fig. 3(d), we didn’t see obvious white patches in the surrounding area of blood injection position. If there are marked edema, it will be imaged in white in the surrounding area of blood injection position.

## Conclusion

The relationship between the CSF and MIPS measurements in hematoma expansion has been investigated in this paper. The MIPS significantly correlates with the CSF and injected blood in hematoma expansion monitoring in a rabbit ICH model. The CSF correlates with the MIPS more tightly than injected blood. There is a reversal point for the MIPS in hematoma expansion monitoring, which is close to the inflection point of the CSF change, which corresponds to the end of CSF compensation. Before the reversal point, the CSF is the leading factor on the MIPS signal. During CSF compensation, CSF reduction compensates for the hematoma expansion in the brain to keep ICP stable. At the same time, the MIPS decreases because of the decreasing CSF volumes. Because of the early physiological responses in the CSF, the MIPS would detect hematoma expansion earlier than ICP. Clinically, the MIPS detects hematoma expansion ahead of ICP and indicates that the end of CSF compensation will be very promising in hematoma expansion monitoring.

## Methods

### Experimental design

**Figure 7:**
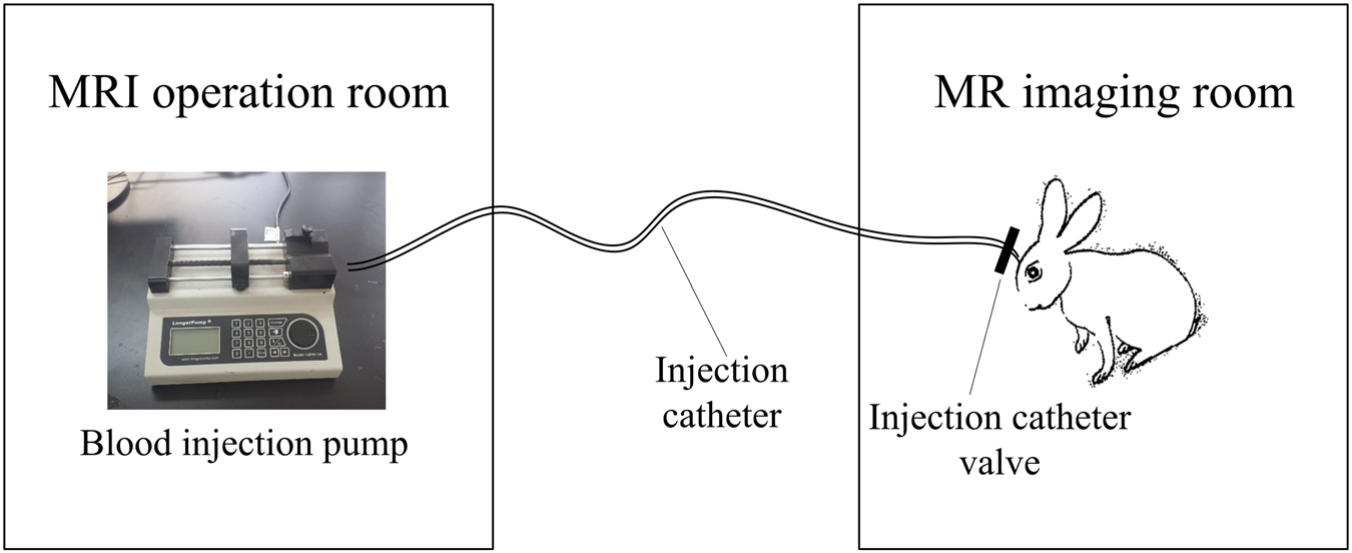
Sketch of the MRI observation experiment on ICH rabbit.

**Figure 8:**
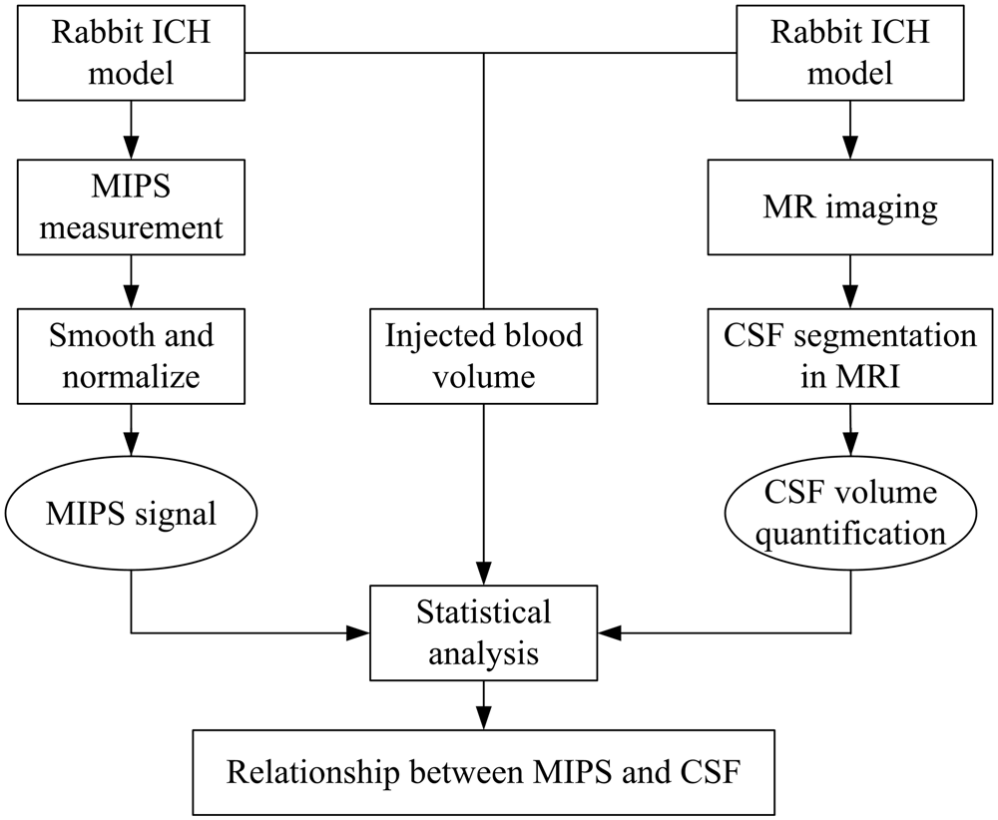
Scheme of Experimental design.

**Figure 9:**
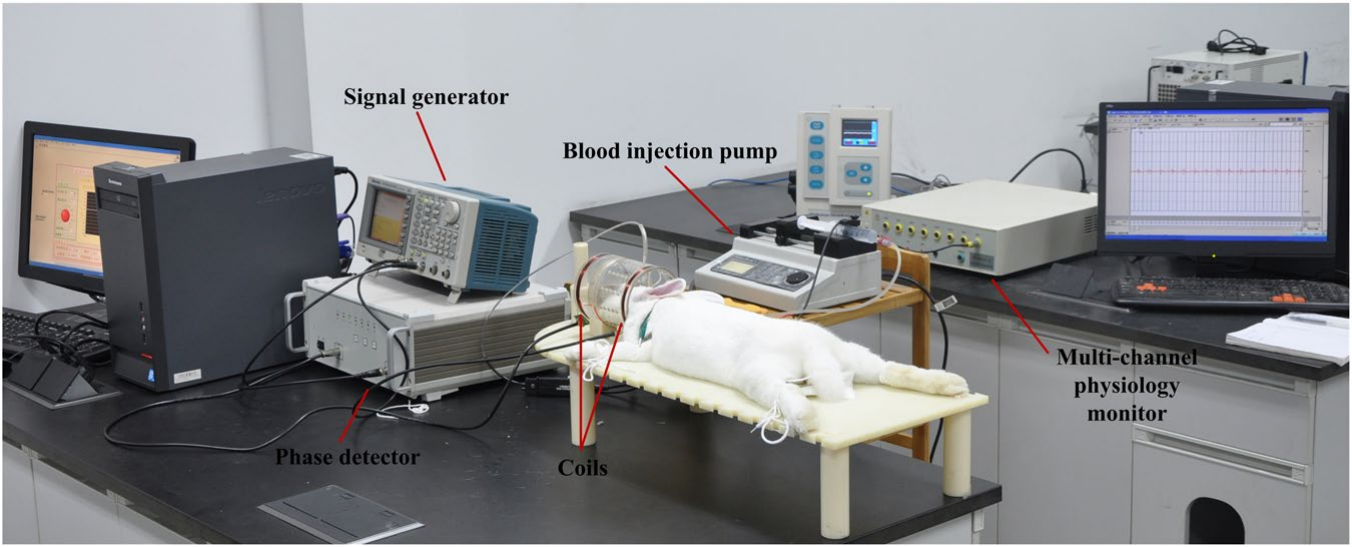
Configuration of MIPS measurement experimental platform.

The main purpose of this paper was to investigate the relationship between the MIPS and CSF in hematoma expansion monitoring. To obtain explicit boundaries between the CSF and surrounding tissues, we utilized MRI to observe changes in CSF during hematoma expansion and quantified the changes in CSF using computer image processing algorithms. The sketch of CSF observation experiments in ICH rabbits by MRI is shown in Fig. 7. The scheme of experimental design is illustrated in Fig. 8. The MIPS signal was collected on ICH rabbits using our self-made MIPS measurement system, as shown in Fig. 9.

In the experiments, the MIPS measurement and the MRI operation proceeded asynchronously because the MIPS measuring electronic equipment could not be placed in the MRI room. During data analysis, the asynchronously measured MIPS and MRI data are synchronized according to the volume of injected blood.

### Rabbit ICH model and Measurements

#### Ethics statement

The Animal Experiments and Ethics Committee of the Third Military Medical University approved all experimental protocols. Animal care was conducted in accordance with the Declaration of Helsinki and the guidelines of the International Association for the Study of Pain (IASP)^16, 17^.

#### ICH model in rabbit

The ICH model^18^ on rabbits is established with autologous blood injection and is describe in our previous work^10^. First, the rabbit is anesthetized by an injection of urethane (25%, 5 ml/kg) in the ear lobe. A total of 3 ml of blood was extracted as the injection autologous blood from the femoral vein. The hair on top of the head was sheared to facilitate cranial drilling. After locating the brain “cross stitch” intersection, a puncture needle point was inserted 6 mm to the right of the coronal suture and 1 mm posterior to the sagittal suture^10^. A needle tube with a diameter of 0.7 mm was inserted into the drilled hole to a depth of 13 mm. Dental cement was used to seal the space around the needle tube adjacent to the cranial hole to prevent the injected blood from flowing along the needle. An extracted 3 ml of blood is injected into the brain at a steady velocity in 54 minutes (Injection speed of 0.056 ml/min).

#### MIPS measurement

The adopted MIPS measurement system is constructed as shown in Fig. 9. The set up consisted mainly of a signal generator, a phase detector, coils, a blood injection pump, a multi-channel physiology monitor instrument (RM6280C, Chengdu Instrument Factory, China) and the MIPS data acquisition software system.

A dual channel arbitrary/function generator (AFG 3252, Tektronix, USA) was set as the signal generator. The phase detector was constructed in house. The excitation coil and sensor coil are made of 1-mm diameter copper twisted 10 times and inserted in a suitable cylindrical plexiglass mold. The two coils have the same radii, R = 5.2 cm, and are separated by a distance of 10.5 cm. The rabbit’s head is inserted into the plexiglass to the point half way between the two coils.

In experiments, the respiration and the vasculature changes in brain caused by cardiac cycle make MIPS signal changes along with the respiration cycle and cardiac cycle. But they are local and periodic changes compared to the concerned change tendency of MIPS in hematoma expansion monitoring. In this study, to investigate the change tendency of MIPS in hematoma expansion monitoring, the measured MIPS signal is smoothed to eliminate the transient fluctuation.

#### CSF quantification from MRIs

MRI of the rabbit brain was carried out on a 3.0 T MRI scanner (Magnetom Spectra with A Tim + Dot System, Siemens, Germany) using an extremity 18 knee coil at Southwest Hospital, Chongqing, China. The sampling perfection with application optimized contrast using different flip-angle evolution (SPACE) sequence was adopted for magnetic resonance imaging. The SPACE sequence is a T2-weighted turbo-spin-echo sequence that can clearly discriminate CSF from the surrounding tissues. The parameters of MRI scanner were set as follows: TR = 1300 ms, ETL = 49, TE = 44 ms, matrix = 320 × 275, FOV = 160 mm × 160 mm, number of slices = 192, slice thickness = 0.5 mm, and slice spacing = 0 mm.

**Figure 10:**
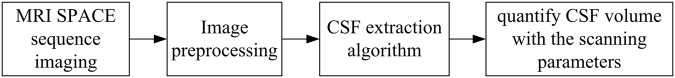
MRI processing and CSF quantification procedure.

**Figure 11:**
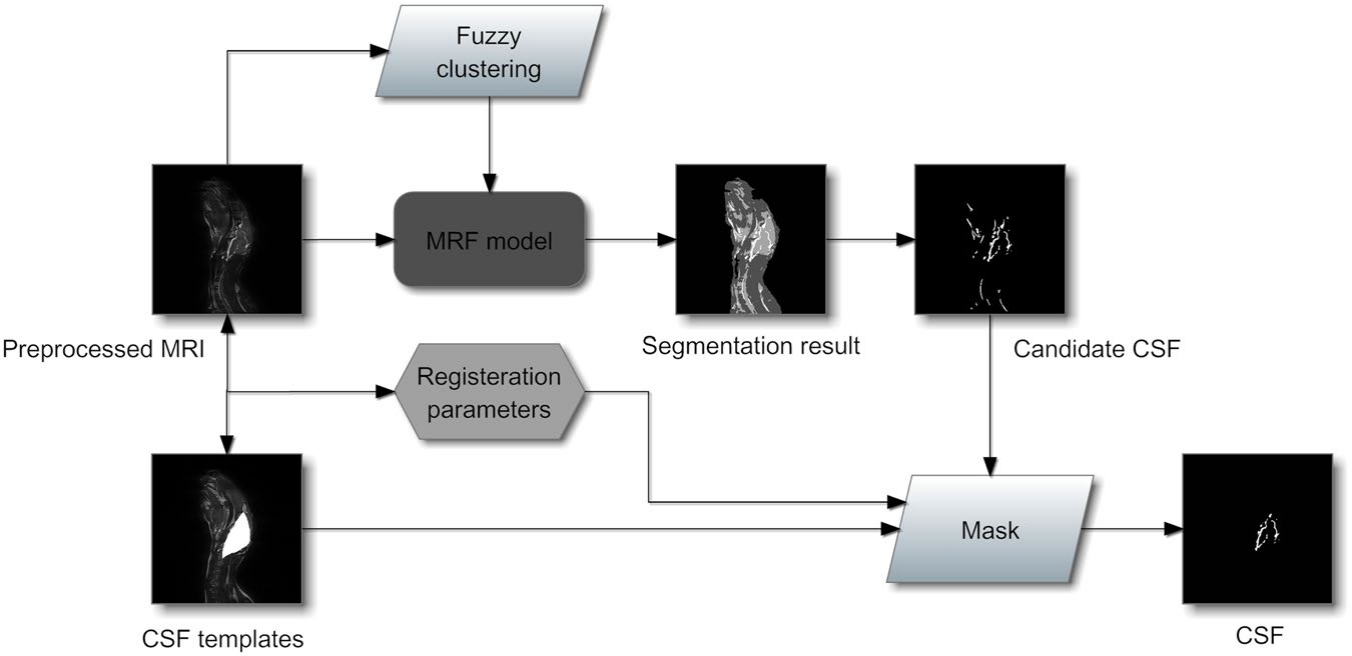
CSF extraction procedure from rabbit brain MRI.

**Figure 12:**
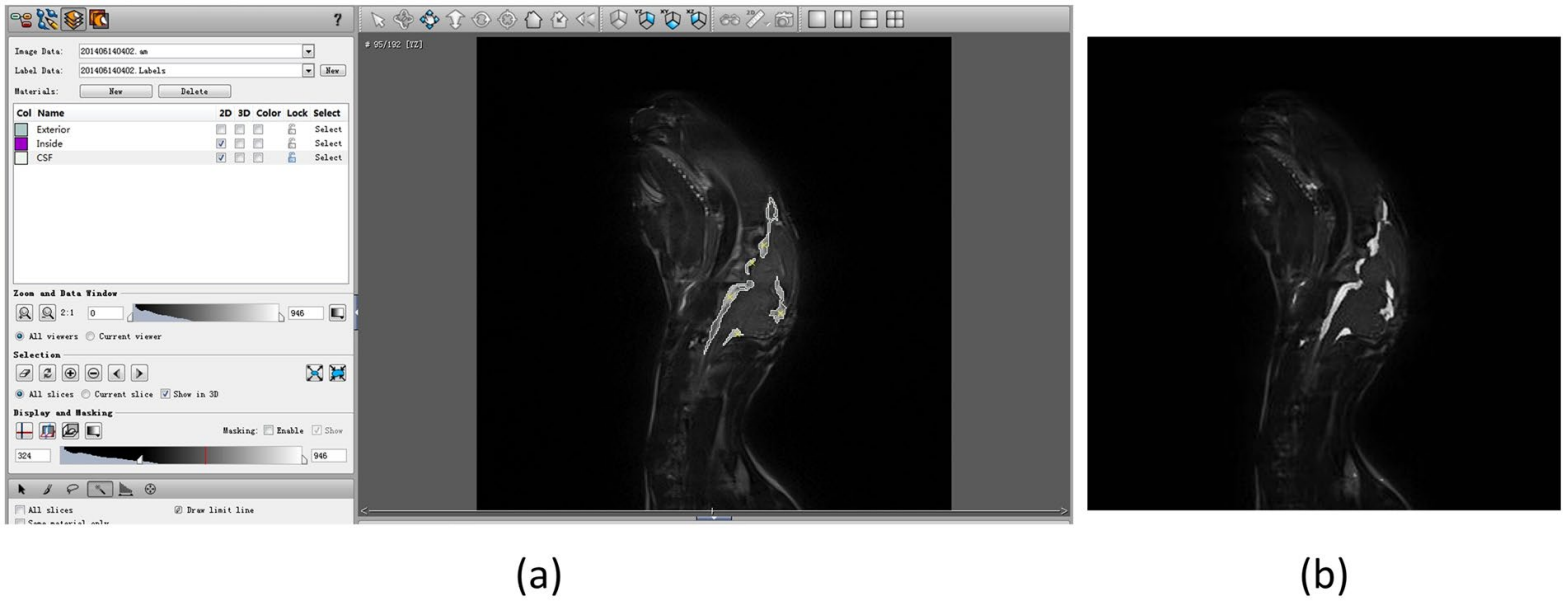
CSF quantification experiment on rabbit, (**a**) segmentation result by manual works in Amira software and (**b**) segmentation result based on the algorithm proposed in previous work.

The quantified CSF volume from the acquired images were retrieved via computer image processing. The processing procedure is shown in Fig. 10. In the image processing procedure, CSF extraction is a critical step. CSF quantification from the acquired images usually suffers due to low resolution, noise pollution, partial volume effect, etc. Therefore, an anti-noise, precise and robust targeting to the rabbit CSF extraction algorithm is desired. In our previous work, a CSF segmentation method from MRI combining the fuzzy clustering and Markov random field was proposed, and it was tested in the noised image databases and comparison experiments (data ready awaiting publication). The candidate CSF is acquired using the segmentation algorithm and is masked with the corresponding prepared rabbit CSF template to derive the exact CSF of the rabbit. Figure 11 shows the CSF data extraction process map for obtaining CSF data from rabbit brain MRI. The CSF quantification experiments are performed on rabbit based on the segmentation algorithm. The segmentation results for the 95th slice in the sagittal direction from the rabbit brain MR image volume by manual works and the proposed algorithm are shown in Fig. 12. The two tissue images show the quantify of CSF, and the amounts of CSF between the two images are very close. In this experiment, we quantified the CSF volume, which was 1.18 ml, manually from the whole MR image volume of the rabbit and retrieved a CSF volume of 1.10 ml by quantification based on the proposed CSF extraction method (the CSF in the vertebral canal outside of the brain was not included). The error between the manual and computer calculations was 6.8% and was considered acceptable for our CSF quantification work.
